# 3-(2,4-Dimeth­oxy­anilino)-8-meth­oxy­dibenz[*b*,*e*]oxepin-11(6*H*)-one

**DOI:** 10.1107/S1600536811002637

**Published:** 2011-01-26

**Authors:** Benjamin Baur, Dieter Schollmeyer, Stefan Laufer

**Affiliations:** a Institute of Pharmacy, Department of Pharmaceutical Chemistry, Eberhard Karls University Tübingen, Auf der Morgenstelle 8, 72076 Tübingen, Germany; b Department of Organic Chemistry, Johannes Gutenberg-University Mainz, Duessbergweg 10-14, 55099 Mainz, Germany

## Abstract

In the title compound, C_23_H_21_NO_5_, the two benzene rings of the tricyclic unit are oriented at a dihedral angle of 37.5 (8)°. The 2,4-dimeth­oxy­anilino residue is oriented at a dihedral angle of 60.2 (8)° towards the phen­oxy ring. In the crystal, the central carbonyl O atom accepts two hydrogen bonds from the N—H and C—H groups. A further inter­molecular C—H⋯O inter­action involving one of the meth­oxy O atoms is also observed.

## Related literature

For palladium-catalysed amination reactions of aryl halides with anilines, see: Jensen *et al.* (2004 ▶). For p38 MAP kinase inhibitors based on dibenzo[*b*,*e*]oxepin-11(6*H*)-one, see: Laufer *et al.* (2006 ▶). 
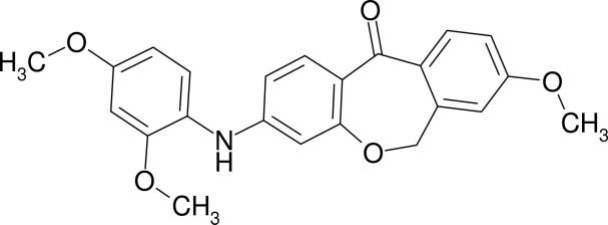


## Experimental

### 

#### Crystal data

C_23_H_21_NO_5_
*M* *_r_* = 391.41
Monoclinic, 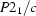

*a* = 9.3277 (9) Å
*b* = 25.8290 (8) Å
*c* = 7.9519 (6) Å
β = 98.914 (3)°
*V* = 1892.7 (2) Å^3^
*Z* = 4
Cu *K*α radiation
μ = 0.80 mm^−1^
*T* = 193 K
0.50 × 0.10 × 0.10 mm

#### Data collection

Enraf–Nonius CAD-4 diffractometer
3847 measured reflections
3578 independent reflections
3041 reflections with *I* > 2σ(*I*)
*R*_int_ = 0.021
3 standard reflections every 60 min  intensity decay: 1%

#### Refinement

*R*[*F* ^2^ > 2σ(*F* ^2^)] = 0.042
*wR*(*F* ^2^) = 0.111
*S* = 1.06
3578 reflections
265 parameters
H-atom parameters constrained
Δρ_max_ = 0.19 e Å^−3^
Δρ_min_ = −0.25 e Å^−3^

### 

Data collection: *CAD-4 Software* (Enraf–Nonius, 1989 ▶); cell refinement: *CAD-4 Software*; data reduction: *CORINC* (Dräger & Gattow, 1971 ▶); program(s) used to solve structure: *SIR97* (Altomare *et al.*, 1999 ▶); program(s) used to refine structure: *SHELXL97* (Sheldrick, 2008 ▶); molecular graphics: *PLATON* (Spek, 2009 ▶); software used to prepare material for publication: *PLATON*.

## Supplementary Material

Crystal structure: contains datablocks I, global. DOI: 10.1107/S1600536811002637/nc2218sup1.cif

Structure factors: contains datablocks I. DOI: 10.1107/S1600536811002637/nc2218Isup2.hkl

Additional supplementary materials:  crystallographic information; 3D view; checkCIF report

## Figures and Tables

**Table 1:** Hydrogen-bond geometry (Å, °)

*D*—H⋯*A*	*D*—H	H⋯*A*	*D*⋯*A*	*D*—H⋯*A*
N12—H12⋯O23^i^	0.87	2.08	2.9403 (18)	168
C4—H4⋯O23^i^	0.95	2.57	3.3000 (19)	134
C20—H20*B*⋯O21^ii^	0.98	2.56	3.496 (3)	160

Symmetry codes: (i) 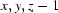; (ii) 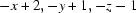.

**Fig. 1.**
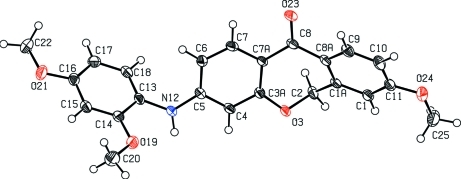
Crystal structure of the title compound with labelling and displacement ellipsoids are drawn at the 50% probability level.

## supplementary crystallographic information

### Comment

Based on dibenzo[b,e]oxepin-11(6*H*)-one (Laufer *et al.* 2006) as novel p38 MAP kinase inhibitors, our intent was to synthesize new oxepin derivatives. The title compound was synthesized in the course of an ongoing study to insert hydrophilic residues at position 8. The two phenyl rings of the tricyclid unit are oriented at a dihedral angle of 37.5 (8°). The 2,4-dimethoxyphenylamino residue is oriented at a dihedral angel of 60.2 (8°) towards the phenoxy ring. The crystal stucture is characterized by several hydrogen bonds. The central carboxyl group O(23) forms two hydrogen bonds towards N(12)—H (2.08 Å) and C(4)—H (2.57 Å) and O(21) forms a hydrogen bond towards C(20)—H (2.56 Å) (Tab. 1).

### Experimental

The preparation of the title compound was achieved by using a palladium catalyzed amination reaction (Jensen et *al.* (2004)).

A mixture of 200 mg (0.73 mmol) 3-chloro-9-methoxy-dibenzo[b,e]oxepin-11(6*H*)-one, 120 mg (0.78 mmol) 2–4-dimethoxyaniline, 1.10 g (3.37 mmol) Cs_2_CO_3_, 45 mg (0.10 mmol) 2-(dicyclohexylphosphino)-2`-4`-6`-triisopropylbiphenyl and 20 mg (0.09 mmol) Pd(OAc)_2_ in 2 ml absolute *tert*-butanol and 10 ml absolute 2.4-dioxane was stirred for 1 h at 284 K under an argon atmosphere. The mixture was then filtered and evaporated under pressure. The residue was purified by column chromatography (SiO_2,_*n*-hexane / ethyl acetate 1 + 1). Crystals of the title compound were obtained by slow evaporation of the solvent from a solution of the title compound in diethylether / *n*-hexane.

^1^H NMR (200 MHz, DMSO) δ in p.p.m. 3.75 (s, 3 H), 3.77 (s, 3 H), 3.84 (s, 3 H), 5.10 (s, 2 H), 6.11 (d, *J*=2.27 Hz, 1 H), 6.52 (m, 2 H), 6.66 (m, 1 H), 7.07 (m, 3 H), 7.82 (d, *J*=7.96 Hz, 1 H), 7.95 (d, *J*=8.97 Hz, 1 H), 8.17 (s, 1 H)

^13^C NMR (50 MHz, DMSO) δ in p.p.m. 55.7, 55.9, 55.9, 73.4, 100.0, 101.0, 105.1, 109.6, 113.2, 114.6, 116.2, 121.7, 126.7, 132.0, 132.9, 133.6, 138.9, 153.8, 154.9, 158.2, 162.6, 163.1, 185.4

### Refinement

Hydrogen atoms attached to carbons were placed at calculated positions with C—H = 0.95 Å (aromatic) or 0.98–0.99 Å (*sp*^3^ C-atom). The position of the N—H H atom was taken from the difference map. All H atoms were refined in the riding-model approximation with isotropic displacement parameters (set at 1.2–1.5 times of the *U*_eq_ of the parent atom).

### Crystal data

**Table:**

C_23_H_21_NO_5_	*F*(000) = 824
*M**_r_* = 391.41	*D*_x_ = 1.374 Mg m^−^^3^
Monoclinic, *P*2_1_/*c*	Cu *K*α radiation, λ = 1.54178 Å
Hall symbol: -P 2ybc	Cell parameters from 25 reflections
*a* = 9.3277 (9) Å	θ = 61–68°
*b* = 25.8290 (8) Å	µ = 0.80 mm^−^^1^
*c* = 7.9519 (6) Å	*T* = 193 K
β = 98.914 (3)°	Needle, yellow
*V* = 1892.7 (2) Å^3^	0.50 × 0.10 × 0.10 mm
*Z* = 4	

### Data collection

**Table:**

Enraf–Nonius CAD-4 diffractometer	*R*_int_ = 0.021
Radiation source: rotating anode	θ_max_ = 69.9°, θ_min_ = 3.4°
graphite	*h* = −11→11
ω/2θ scans	*k* = 0→31
3847 measured reflections	*l* = 0→9
3578 independent reflections	3 standard reflections every 60 min
3041 reflections with *I* > 2σ(*I*)	intensity decay: 1%

### Refinement

**Table:**

Refinement on *F*^2^	Primary atom site location: structure-invariant direct methods
Least-squares matrix: full	Secondary atom site location: difference Fourier map
*R*[*F*^2^ > 2σ(*F*^2^)] = 0.042	Hydrogen site location: inferred from neighbouring sites
*wR*(*F*^2^) = 0.111	H-atom parameters constrained
*S* = 1.06	*w* = 1/[σ^2^(*F*_o_^2^) + (0.0517*P*)^2^ + 0.6681*P*] where *P* = (*F*_o_^2^ + 2*F*_c_^2^)/3
3578 reflections	(Δ/σ)_max_ < 0.001
265 parameters	Δρ_max_ = 0.19 e Å^−^^3^
0 restraints	Δρ_min_ = −0.25 e Å^−^^3^

### Special details

**Table:**

Geometry. All e.s.d.'s (except the e.s.d. in the dihedral angle between two l.s. planes) are estimated using the full covariance matrix. The cell e.s.d.'s are taken into account individually in the estimation of e.s.d.'s in distances, angles and torsion angles; correlations between e.s.d.'s in cell parameters are only used when they are defined by crystal symmetry. An approximate (isotropic) treatment of cell e.s.d.'s is used for estimating e.s.d.'s involving l.s. planes.
Refinement. Refinement of *F*^2^ against ALL reflections. The weighted *R*-factor *wR* and goodness of fit *S* are based on *F*^2^, conventional *R*-factors *R* are based on *F*, with *F* set to zero for negative *F*^2^. The threshold expression of *F*^2^ > σ(*F*^2^) is used only for calculating *R*-factors(gt) *etc*. and is not relevant to the choice of reflections for refinement. *R*-factors based on *F*^2^ are statistically about twice as large as those based on *F*, and *R*- factors based on ALL data will be even larger.

### Fractional atomic coordinates and isotropic or equivalent isotropic displacement parameters (Å^2^)

**Table:**

	*x*	*y*	*z*	*U*_iso_*/*U*_eq_	
C1	0.02652 (18)	0.28134 (6)	0.3263 (2)	0.0231 (3)	
H1	−0.0153	0.2542	0.2545	0.028*	
C1A	0.16914 (17)	0.29698 (6)	0.32152 (19)	0.0203 (3)	
C2	0.25880 (19)	0.26887 (6)	0.2104 (2)	0.0239 (4)	
H2A	0.3481	0.2556	0.2807	0.029*	
H2B	0.2035	0.2389	0.1567	0.029*	
O3	0.29732 (12)	0.30244 (5)	0.08016 (13)	0.0249 (3)	
C3A	0.41536 (17)	0.33353 (6)	0.1250 (2)	0.0196 (3)	
C4	0.48724 (17)	0.34554 (6)	−0.0096 (2)	0.0211 (3)	
H4	0.4503	0.3328	−0.1199	0.025*	
C5	0.61280 (17)	0.37589 (6)	0.01266 (19)	0.0206 (3)	
C6	0.66950 (18)	0.39254 (6)	0.1786 (2)	0.0231 (3)	
H6	0.7579	0.4114	0.1991	0.028*	
C7	0.59571 (18)	0.38115 (6)	0.3098 (2)	0.0239 (3)	
H7	0.6347	0.3931	0.4205	0.029*	
C7A	0.46456 (17)	0.35261 (6)	0.29013 (19)	0.0197 (3)	
C8	0.38732 (18)	0.35207 (6)	0.43906 (19)	0.0220 (3)	
C8A	0.23163 (18)	0.33692 (6)	0.42594 (19)	0.0212 (3)	
C9	0.14745 (19)	0.36140 (7)	0.5343 (2)	0.0266 (4)	
H9	0.1886	0.3887	0.6061	0.032*	
C10	0.00634 (19)	0.34659 (7)	0.5384 (2)	0.0279 (4)	
H10	−0.0498	0.3642	0.6104	0.034*	
C11	−0.05416 (18)	0.30576 (7)	0.4367 (2)	0.0241 (4)	
N12	0.67328 (15)	0.38860 (6)	−0.12843 (17)	0.0258 (3)	
H12	0.6152	0.3845	−0.2242	0.031*	
C13	0.80880 (17)	0.41301 (6)	−0.12979 (19)	0.0217 (3)	
C14	0.81422 (17)	0.45637 (6)	−0.2367 (2)	0.0220 (3)	
C15	0.94631 (18)	0.47876 (6)	−0.2511 (2)	0.0240 (4)	
H15	0.9501	0.5074	−0.3251	0.029*	
C16	1.07366 (17)	0.45964 (6)	−0.1579 (2)	0.0233 (4)	
C17	1.07033 (18)	0.41758 (7)	−0.0502 (2)	0.0260 (4)	
H17	1.1571	0.4047	0.0145	0.031*	
C18	0.93686 (19)	0.39460 (7)	−0.0391 (2)	0.0256 (4)	
H18	0.9340	0.3654	0.0330	0.031*	
O19	0.68401 (12)	0.47311 (5)	−0.32059 (16)	0.0289 (3)	
C20	0.6874 (2)	0.51958 (8)	−0.4183 (3)	0.0404 (5)	
H20A	0.5880	0.5303	−0.4631	0.061*	
H20B	0.7408	0.5132	−0.5132	0.061*	
H20C	0.7357	0.5471	−0.3456	0.061*	
O21	1.19612 (13)	0.48548 (5)	−0.18470 (17)	0.0333 (3)	
C22	1.33194 (19)	0.46751 (9)	−0.0997 (3)	0.0386 (5)	
H22A	1.3330	0.4691	0.0237	0.058*	
H22B	1.4097	0.4894	−0.1306	0.058*	
H22C	1.3469	0.4317	−0.1333	0.058*	
O23	0.44763 (14)	0.36837 (5)	0.57752 (14)	0.0338 (3)	
O24	−0.19058 (13)	0.29162 (5)	0.45756 (16)	0.0319 (3)	
C25	−0.2614 (2)	0.25277 (8)	0.3469 (3)	0.0399 (5)	
H25A	−0.2687	0.2644	0.2285	0.060*	
H25B	−0.3589	0.2466	0.3741	0.060*	
H25C	−0.2052	0.2206	0.3619	0.060*	

### Atomic displacement parameters (Å^2^)

**Table:**

	*U*^11^	*U*^22^	*U*^33^	*U*^12^	*U*^13^	*U*^23^
C1	0.0268 (8)	0.0244 (8)	0.0184 (8)	−0.0034 (7)	0.0049 (6)	−0.0003 (6)
C1A	0.0267 (8)	0.0212 (8)	0.0136 (7)	−0.0013 (6)	0.0051 (6)	0.0025 (6)
C2	0.0306 (9)	0.0223 (8)	0.0212 (8)	−0.0073 (7)	0.0117 (7)	−0.0024 (6)
O3	0.0293 (6)	0.0327 (6)	0.0140 (5)	−0.0121 (5)	0.0074 (5)	−0.0038 (5)
C3A	0.0211 (8)	0.0202 (7)	0.0183 (7)	−0.0006 (6)	0.0053 (6)	−0.0005 (6)
C4	0.0262 (8)	0.0235 (8)	0.0141 (7)	−0.0010 (6)	0.0049 (6)	−0.0008 (6)
C5	0.0242 (8)	0.0209 (8)	0.0176 (7)	0.0007 (6)	0.0060 (6)	0.0026 (6)
C6	0.0239 (8)	0.0251 (8)	0.0204 (8)	−0.0050 (6)	0.0042 (6)	0.0000 (6)
C7	0.0278 (8)	0.0278 (8)	0.0154 (7)	−0.0023 (7)	0.0015 (6)	−0.0013 (6)
C7A	0.0231 (8)	0.0227 (8)	0.0136 (7)	−0.0014 (6)	0.0039 (6)	0.0012 (6)
C8	0.0297 (9)	0.0223 (8)	0.0140 (7)	−0.0027 (7)	0.0038 (6)	0.0010 (6)
C8A	0.0281 (8)	0.0228 (8)	0.0138 (7)	−0.0014 (7)	0.0067 (6)	0.0017 (6)
C9	0.0370 (9)	0.0234 (8)	0.0211 (8)	−0.0025 (7)	0.0099 (7)	−0.0032 (7)
C10	0.0326 (9)	0.0279 (9)	0.0266 (9)	0.0039 (7)	0.0148 (7)	−0.0013 (7)
C11	0.0245 (8)	0.0281 (9)	0.0209 (8)	0.0026 (7)	0.0072 (7)	0.0053 (6)
N12	0.0259 (7)	0.0372 (8)	0.0147 (7)	−0.0093 (6)	0.0045 (5)	0.0017 (6)
C13	0.0256 (8)	0.0255 (8)	0.0158 (7)	−0.0039 (7)	0.0086 (6)	−0.0028 (6)
C14	0.0234 (8)	0.0259 (8)	0.0175 (7)	0.0004 (6)	0.0055 (6)	−0.0015 (6)
C15	0.0285 (9)	0.0231 (8)	0.0213 (8)	−0.0027 (7)	0.0071 (7)	0.0017 (6)
C16	0.0235 (8)	0.0255 (8)	0.0224 (8)	−0.0029 (7)	0.0079 (7)	−0.0048 (6)
C17	0.0254 (8)	0.0292 (9)	0.0229 (8)	0.0020 (7)	0.0021 (7)	−0.0003 (7)
C18	0.0317 (9)	0.0267 (9)	0.0192 (8)	−0.0012 (7)	0.0071 (7)	0.0036 (7)
O19	0.0242 (6)	0.0307 (7)	0.0312 (7)	−0.0013 (5)	0.0026 (5)	0.0085 (5)
C20	0.0358 (10)	0.0361 (11)	0.0467 (12)	0.0000 (8)	−0.0015 (9)	0.0184 (9)
O21	0.0220 (6)	0.0365 (7)	0.0416 (7)	−0.0069 (5)	0.0053 (5)	0.0051 (6)
C22	0.0219 (9)	0.0518 (12)	0.0410 (11)	−0.0060 (8)	0.0010 (8)	0.0010 (9)
O23	0.0369 (7)	0.0508 (8)	0.0140 (6)	−0.0133 (6)	0.0041 (5)	−0.0057 (5)
O24	0.0264 (6)	0.0390 (7)	0.0327 (7)	−0.0023 (5)	0.0120 (5)	0.0003 (6)
C25	0.0294 (10)	0.0460 (12)	0.0459 (12)	−0.0084 (9)	0.0108 (9)	−0.0038 (10)

### Geometric parameters (Å, °)

**Table:**

C1—C11	1.393 (2)	C11—O24	1.358 (2)
C1—C1A	1.396 (2)	N12—C13	1.414 (2)
C1—H1	0.9500	N12—H12	0.8699
C1A—C8A	1.394 (2)	C13—C18	1.381 (2)
C1A—C2	1.496 (2)	C13—C14	1.412 (2)
C2—O3	1.4382 (19)	C14—O19	1.362 (2)
C2—H2A	0.9900	C14—C15	1.382 (2)
C2—H2B	0.9900	C15—C16	1.390 (2)
O3—C3A	1.3648 (19)	C15—H15	0.9500
C3A—C4	1.383 (2)	C16—O21	1.3680 (19)
C3A—C7A	1.411 (2)	C16—C17	1.386 (2)
C4—C5	1.398 (2)	C17—C18	1.394 (2)
C4—H4	0.9500	C17—H17	0.9500
C5—N12	1.371 (2)	C18—H18	0.9500
C5—C6	1.411 (2)	O19—C20	1.433 (2)
C6—C7	1.368 (2)	C20—H20A	0.9800
C6—H6	0.9500	C20—H20B	0.9800
C7—C7A	1.416 (2)	C20—H20C	0.9800
C7—H7	0.9500	O21—C22	1.418 (2)
C7A—C8	1.479 (2)	C22—H22A	0.9800
C8—O23	1.2304 (19)	C22—H22B	0.9800
C8—C8A	1.492 (2)	C22—H22C	0.9800
C8A—C9	1.403 (2)	O24—C25	1.428 (2)
C9—C10	1.376 (2)	C25—H25A	0.9800
C9—H9	0.9500	C25—H25B	0.9800
C10—C11	1.394 (2)	C25—H25C	0.9800
C10—H10	0.9500		
			
C11—C1—C1A	119.75 (15)	O24—C11—C10	115.82 (15)
C11—C1—H1	120.1	C1—C11—C10	119.89 (15)
C1A—C1—H1	120.1	C5—N12—C13	126.48 (14)
C8A—C1A—C1	120.67 (15)	C5—N12—H12	114.1
C8A—C1A—C2	119.38 (14)	C13—N12—H12	118.9
C1—C1A—C2	119.87 (14)	C18—C13—C14	118.62 (15)
O3—C2—C1A	110.96 (13)	C18—C13—N12	122.99 (15)
O3—C2—H2A	109.4	C14—C13—N12	118.28 (14)
C1A—C2—H2A	109.4	O19—C14—C15	124.42 (15)
O3—C2—H2B	109.4	O19—C14—C13	115.74 (14)
C1A—C2—H2B	109.4	C15—C14—C13	119.83 (15)
H2A—C2—H2B	108.0	C14—C15—C16	120.43 (15)
C3A—O3—C2	116.61 (12)	C14—C15—H15	119.8
O3—C3A—C4	113.44 (14)	C16—C15—H15	119.8
O3—C3A—C7A	125.57 (14)	O21—C16—C17	125.29 (15)
C4—C3A—C7A	120.99 (14)	O21—C16—C15	114.13 (15)
C3A—C4—C5	121.68 (15)	C17—C16—C15	120.58 (15)
C3A—C4—H4	119.2	C16—C17—C18	118.58 (16)
C5—C4—H4	119.2	C16—C17—H17	120.7
N12—C5—C4	118.28 (14)	C18—C17—H17	120.7
N12—C5—C6	123.38 (14)	C13—C18—C17	121.93 (15)
C4—C5—C6	118.33 (14)	C13—C18—H18	119.0
C7—C6—C5	119.20 (15)	C17—C18—H18	119.0
C7—C6—H6	120.4	C14—O19—C20	116.01 (13)
C5—C6—H6	120.4	O19—C20—H20A	109.5
C6—C7—C7A	123.77 (15)	O19—C20—H20B	109.5
C6—C7—H7	118.1	H20A—C20—H20B	109.5
C7A—C7—H7	118.1	O19—C20—H20C	109.5
C3A—C7A—C7	115.82 (14)	H20A—C20—H20C	109.5
C3A—C7A—C8	127.84 (14)	H20B—C20—H20C	109.5
C7—C7A—C8	115.86 (14)	C16—O21—C22	118.15 (14)
O23—C8—C7A	120.10 (15)	O21—C22—H22A	109.5
O23—C8—C8A	117.22 (14)	O21—C22—H22B	109.5
C7A—C8—C8A	122.43 (13)	H22A—C22—H22B	109.5
C1A—C8A—C9	118.50 (15)	O21—C22—H22C	109.5
C1A—C8A—C8	123.26 (14)	H22A—C22—H22C	109.5
C9—C8A—C8	118.04 (14)	H22B—C22—H22C	109.5
C10—C9—C8A	121.18 (16)	C11—O24—C25	117.80 (14)
C10—C9—H9	119.4	O24—C25—H25A	109.5
C8A—C9—H9	119.4	O24—C25—H25B	109.5
C9—C10—C11	119.97 (15)	H25A—C25—H25B	109.5
C9—C10—H10	120.0	O24—C25—H25C	109.5
C11—C10—H10	120.0	H25A—C25—H25C	109.5
O24—C11—C1	124.26 (16)	H25B—C25—H25C	109.5
			
C11—C1—C1A—C8A	−0.2 (2)	C7A—C8—C8A—C9	−146.90 (16)
C11—C1—C1A—C2	176.53 (15)	C1A—C8A—C9—C10	0.0 (2)
C8A—C1A—C2—O3	−66.78 (19)	C8—C8A—C9—C10	−174.98 (15)
C1—C1A—C2—O3	116.48 (16)	C8A—C9—C10—C11	1.6 (3)
C1A—C2—O3—C3A	83.27 (17)	C1A—C1—C11—O24	−176.31 (15)
C2—O3—C3A—C4	151.24 (14)	C1A—C1—C11—C10	1.8 (2)
C2—O3—C3A—C7A	−28.6 (2)	C9—C10—C11—O24	175.75 (15)
O3—C3A—C4—C5	−178.36 (14)	C9—C10—C11—C1	−2.5 (3)
C7A—C3A—C4—C5	1.5 (2)	C4—C5—N12—C13	−171.32 (15)
C3A—C4—C5—N12	−176.34 (15)	C6—C5—N12—C13	9.7 (3)
C3A—C4—C5—C6	2.7 (2)	C5—N12—C13—C18	53.5 (2)
N12—C5—C6—C7	175.15 (16)	C5—N12—C13—C14	−130.35 (17)
C4—C5—C6—C7	−3.8 (2)	C18—C13—C14—O19	−179.03 (14)
C5—C6—C7—C7A	0.9 (3)	N12—C13—C14—O19	4.6 (2)
O3—C3A—C7A—C7	175.56 (15)	C18—C13—C14—C15	1.2 (2)
C4—C3A—C7A—C7	−4.3 (2)	N12—C13—C14—C15	−175.09 (14)
O3—C3A—C7A—C8	−12.8 (3)	O19—C14—C15—C16	178.75 (15)
C4—C3A—C7A—C8	167.39 (16)	C13—C14—C15—C16	−1.5 (2)
C6—C7—C7A—C3A	3.1 (2)	C14—C15—C16—O21	179.95 (15)
C6—C7—C7A—C8	−169.56 (16)	C14—C15—C16—C17	0.6 (2)
C3A—C7A—C8—O23	176.92 (16)	O21—C16—C17—C18	−178.63 (16)
C7—C7A—C8—O23	−11.4 (2)	C15—C16—C17—C18	0.7 (2)
C3A—C7A—C8—C8A	−9.0 (3)	C14—C13—C18—C17	0.0 (2)
C7—C7A—C8—C8A	162.71 (15)	N12—C13—C18—C17	176.18 (15)
C1—C1A—C8A—C9	−0.7 (2)	C16—C17—C18—C13	−1.0 (3)
C2—C1A—C8A—C9	−177.46 (15)	C15—C14—O19—C20	−5.2 (2)
C1—C1A—C8A—C8	173.99 (15)	C13—C14—O19—C20	175.09 (16)
C2—C1A—C8A—C8	−2.7 (2)	C17—C16—O21—C22	1.6 (3)
O23—C8—C8A—C1A	−147.38 (16)	C15—C16—O21—C22	−177.76 (16)
C7A—C8—C8A—C1A	38.3 (2)	C1—C11—O24—C25	−6.7 (2)
O23—C8—C8A—C9	27.4 (2)	C10—C11—O24—C25	175.15 (16)

### Hydrogen-bond geometry (Å, °)

**Table:**

*D*—H···*A*	*D*—H	H···*A*	*D*···*A*	*D*—H···*A*
N12—H12···O23^i^	0.87	2.08	2.9403 (18)	168
C4—H4···O23^i^	0.95	2.57	3.3000 (19)	134
C20—H20B···O21^ii^	0.98	2.56	3.496 (3)	160

Symmetry codes: (i) *x*, *y*, *z*−1; (ii) −*x*+2, −*y*+1, −*z*−1.
